# Targeting the small airways with dry powder adenosine: a challenging concept

**DOI:** 10.1080/20018525.2017.1369328

**Published:** 2017-09-06

**Authors:** Erica van der Wiel, Anne J. Lexmond, Maarten van den Berge, Dirkje S. Postma, Paul Hagedoorn, Henderik W. Frijlink, Martijn P. Farenhorst, Anne H. de Boer, Nick H. T. ten Hacken

**Affiliations:** ^a^ Department of Pulmonary Medicine, University of Groningen, University Medical Center Groningen, Groningen, the Netherlands; ^b^ Groningen Research Institute for Asthma and COPD (GRIAC), University of Groningen, University Medical Center Groningen, Groningen, the Netherlands; ^c^ Department of Pharmaceutical Technology and Biopharmacy, University of Groningen, Groningen, the Netherlands; ^d^ Lung Function Department, University Medical Center Groningen, Groningen, the Netherlands

**Keywords:** Asthma, bronchial hyperresponsiveness, small airways, bronchoprovocation test, adenosine

## Abstract

**Background**: Small-particle inhaled corticosteroids (ICS) provide a higher small airway deposition than large-particle ICS. However, we are still not able to identify asthma patients who will profit most from small-particle treatment.

**Objective**: We aimed to identify these patients by selectively challenging the small and large airways. We hypothesized that the airways could be challenged selectively using small- and large-particle adenosine, both inhaled at a high and a low flow rate.

**Design**: In this cross-over study 11 asthma subjects performed four dry powder adenosine tests, with either small (MMAD 2.7 µm) or large (MMAD 6.0 µm) particles, inhaled once with a low flow rate (30 l min^–1^) and once with a high flow rate (60 l min^–1^). Spirometry and impulse oscillometry were performed after every bronchoprovocation step. We assumed that FEV_1_ reflects the large airways, and FEF_25–75%_, R5-R20 and X5 reflect the small airways.

**Results**: The four adenosine tests were not significantly different with respect to the threshold values of FEV_1_ (*p* = 0.12), FEF_25–75%_ (*p* = 0.37), R5-R20 (*p* = 0.60) or X5 (*p* = 0.46). Both small- and large-particle adenosine induced a response in the small airways in the majority of the tests.

**Conclusions**: In contrast to our hypothesis, all four adenosine tests provoked a response in the small airways and we could not identify different large- or small-airway responders. Interestingly, even the test with large particles and a high flow rate induced a small-airway response, suggesting that selective challenging of the small airways is not necessary. Future studies should investigate the relation between particle deposition and the site of an airway response.

## Introduction

Recent studies in asthma suggest that treatment with small-particle inhaled corticosteroids (ICS) associates with improved asthma control and less small airway dysfunction.[1–3] An advantage of small-particle instead of large-particle inhaled treatment could be a higher total lung deposition in addition to better deposition in the small airways.[4,5] Yet, we are currently not able to select asthma patients with small airway dysfunction, i.e. those who will probably benefit most from the inhaled small-particle medication.

Cohen and colleagues tried for the first time to identify responders and non-responders to treatment with small-particle inhaled corticosteroids (ICS) using an indirect bronchoprovocation test with large and small particles of dissolved adenosine 5ʹ-monophosphate (AMP).[6] They saw a significant improvement in the 20% fall in FEV_1_ (PC_20_) after bronchoprovocation with small-particle AMP, and not large-particle AMP, after a 4-week treatment with a small-particle ICS. Interpretation of the data was hampered by the fact that only 60% of the subjects reached a PC_20_ with the small-particle AMP test. The low response rate was partly attributed to the very small mass median aerodynamic diameter (MMAD) of 1.1 μm, which is probably too small for effective airway deposition with a tidal-breathing method. The challenge with nebulized AMP has a number of other disadvantages, i.e. it is impossible to dissolve AMP at higher concentrations than approximately 320 mg ml^–1^, the particle size distribution and nebulizer output rate are not consistent over the entire concentration range, and good clinical manufacturing of sterile, diluted agents is more complicated than manufacturing of most dry powder formulations.[7]

Bronchoprovocation with a dry powder formulation may help to overcome the above-described disadvantages of AMP nebulization.[8]

In the current pilot study, we aimed to challenge the small and large airways selectively with dry powder adenosine. For this challenge, we chose the particle size and inhalation maneuver used by Usmani and colleagues [4] as a reference. These authors studied deposition of monodisperse albuterol aerosol (geometric SD ≤1.22), a bronchodilator instead of a bronchoconstrictor stimulus as we did, after one single inspiration by two-dimensional scintigraphic imaging.[4] They observed a significant improvement of deposition in the small airways when inhaling smaller particles, i.e. a small airway deposition of 10, 17 and 25% with respect to particles with an MMAD of 6, 3 and 1.5 μm. In addition, a slow inspiratory flow increased the deposition in the small airways compared to a high flow rate, showing that small particles inhaled slowly deposit in the small airways, whereas large particles deposit more centrally in the larger airways. In addition, only 5% of the large particles (6 μm) inhaled with a high flow rate (67l min^–1^) deposited in the small airways.

**Figure 1.**
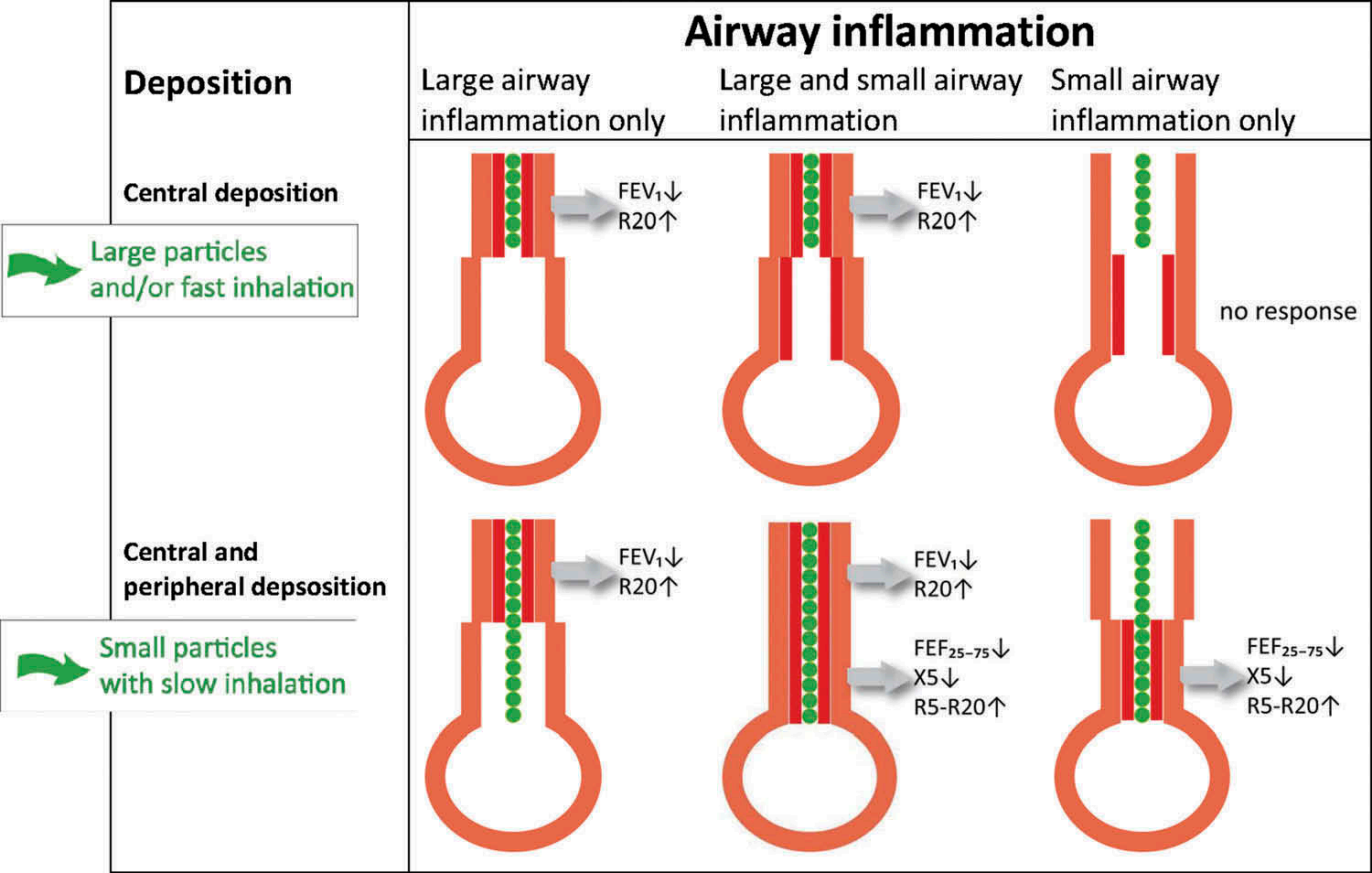
Hypothetical response patterns with dry powder adenosine. This simplified figure shows the hypothetical response patterns upon bronchoprovocation with dry powder adenosine in the present study. The first assumption is that there are three sites of inflammation: isolated large airway inflammation (left picture), both large and small airway inflammation (middle picture), isolated small airway inflammation (right picture). The second assumption is that airways only obstruct if adenosine particles (green) are deposited in inflamed airways (red layer). The third assumption is that large airway dysfunction is reflected by FEV_1_ and R20, and small airway dysfunction by FEF_27–75%_, R5-R20 and X5. The fourth assumption is that large (MMAD 6.0 µm) particles, or particles inhaled with a high flow rate (60–70 l min^–1^) deposit in the central airways (upper row), whereas small (MMAD 2.7 µm) particles inhaled with a low flow rate (30–40 l min^–1^) deposit in the central and peripheral airways (lower row). In this study we had no information about the site of airway inflammation, nor the site of adenosine deposition. If the above-described assumptions are correct there are four potential response patterns (Table 5).

We hypothesized that a small-particle slow-inhalation bronchoprovocation test gives a higher deposition and thus a higher response in the small airways than a test with large particles and/or inhalation with a high flow rate (Figure 1).

## Methods

### Study design

In this cross-over study subjects performed four adenosine bronchoprovocation tests in randomized order. The study was approved by the Medical Ethics Committee of the University Medical Center Groningen and all subjects gave written informed consent (NCT01610921). Based on the results of Usmani and colleagues [4] we aimed to include at least 10 subjects, expecting that we could gather enough data to demonstrate the potential of the principles analyzed in this pilot study.

Subjects with a doctor’s diagnosis of asthma, never smokers, 18–65 years old were recruited via advertisements. Subjects were steroid naive or stopped steroids 4 weeks before the first visit. Exclusion criteria were a recent exacerbation (<2 months), upper respiratory tract infection (<2 weeks), FEV_1_ < 50% predicted or <1.2 l, pregnancy, or a diagnosis of another pulmonary disease. Subjects were characterized during a baseline visit assessing impulse oscillometry (IOS), spirometry, body plethysmography and a conventional nebulized AMP bronchoprovocation test.[9–13] Subjects with a positive AMP response (PC_20_ ≤ 320 mg ml^–1^) on this baseline visit were included.

All adenosine bronchoprovocation tests were scheduled during the same part of the day with an interval between 2 and 14 days. Patients stayed steroid naive during the study and had to withhold short-acting bronchodilators at least 6 hours before each visit. Dry powder adenosine bronchoprovocation tests were carried out with either small (MMAD 2.7 µm, geometric SD of 1.5) or large particles (MMAD 6.0 µm, geometric SD of 1.8). Both tests were performed once with a slow inhalation (30–40 l min^–1^) and once with a fast inhalation (60–70 l min^–1^).

### Adenosine test specifications

The flow rate curve was measured and shown on a computer screen during each inhalation of adenosine in order to give visual feedback and target the desired flow rates. Peak and mean (min–max) flow rates were recorded for every inhalation maneuver. Before starting the tests, subjects practiced the inhalation maneuver until a proper inhalation was performed.

The adenosine dry powder bronchoprovocation test was performed with doubling adenosine doses ranging from 0.04 to 20 mg. Each dose was provided in a single blister, except the 20 mg dose, which was provided in two blisters of 10 mg. After full expiration, subjects inhaled the adenosine with one deep inspiration with the required flow rate, and then held their breath for 10 s. The blisters were directly checked upon dose release and if required, a second inspiration was performed to ensure administration of the full dose. The test inhaler is related to the Twincer™ has an air classifier dispersion system similar to the Novolizer®.[14,15] Performance testing results, specifications of the inhaler in combination with dry powder adenosine, and the first clinical pilot in five asthma patients have been described by Lexmond and colleagues.[8,16]

Adenosine bronchoprovocation took place with a time interval of 3 min and measurements of IOS at 30 s and FVC at 90 s similar to the nebulized AMP bronchoprovocation test. Due to safety reasons the bronchoprovocation test was stopped when the FEV_1_ fell ≥20% compared to baseline.

### Data analysis

A positive response to the bronchoprovocation test was defined as a 20% fall (PD_20_) in the FEV_1_ or FEF_25–75%_ or a 40% increase (PD_40_) in any of the IOS parameters R20, R5-R20 or X5. The 40% increase in IOS parameters was based on previous studies using a threshold of 40% increase in resistance measured with the forced oscillation technique or 40% decrease in specific airway conductance measured with body plethysmography.[9,17,18] FEV_1_ and R20 were considered as large airway parameters, FEF_25–75%_, R5-R20 and X5 as small airway parameters.

The adenosine dose causing a 20% fall (PD_20_) or 40% increase (PD_40_) was calculated with interpolation using the log-transformed doses. If a 20% fall in the spirometric parameters or a 40% increase in the IOS parameters was reached after the first dose (0.04 mg), a PD of 0.02 was noted. If a 20% fall or 40% increase was not reached after the highest dose (20 mg), PD_20_ and PD_40_ were calculated by extrapolation with a maximum of 40 mg. If the test was stopped due to a 20% fall in FEV_1_, extrapolation of PD_20_ for the FEF_25–7%_ or PD_40_ for the IOS parameters was only allowed if the calculated provocative dose was not higher than twice the last given dose, otherwise no PD_20_ or PD_40_ was assigned. PD_20_ and PD_40_ values were not based on outliers and were verified visually.

The Friedman test was used to test for differences between the four adenosine bronchoprovocation methods with respect to eliciting a bronchoconstrictive response in the large and small airways. Linear mixed effect models were used to estimate the effect of particle size and inhalation flow rate on PD_20_ and PD_40_. In addition, linear mixed effect models were used to make pairwise comparisons between the four tests. Analyses were performed with SPSS version 20.

## Results

### Characteristics

A total of 26 subjects gave written informed consent and 11 subjects were included in the study. Fifteen subjects were excluded due to either a severe (PC_20_ < 0.04 mg ml^–1^) or no response to nebulized AMP at screening. One of the included subjects dropped out during the study because of respiratory complaints. This subject performed only two large-particle adenosine tests. All obtained data have been used in the analyses. Baseline characteristics are shown in Table 1.

**Table 1.** Characteristics of study population (*n* = 11).

	Median	(IQrange)
Age (years)	22	(20;40)
Gender (*n*, female)	7	
ICS use (*n*, yes) *	10	
ICS dose (µg)^#^	500	(0;1500)
FEV_1_ (%pred)	92	(86;113)
FEV_1_/FVC (%)	76	(65;97)
FEF_25–75%_ (%pred)	62	(49;120)
RV (%pred)	92	(35;131)
R20 (kPa l^–1^ s^–1^)	0.37	(0.27;0.50)
R5-R20 (kPa l^–1^ s^–1^)	0.04	(−0.02;0.32)
X5 (kPa l^–1^ s^–1^)	−0.1	(−0.25;-0.05)
AX (kPa l^–1^)	0.23	(0.07;2.73)
PC_20_ AMP (mg ml^–1^)	15.3	(1.51;34.8)

*Four weeks before start of the study; ^#^beclomethasone equivalent.

AMP: adenosine 5ʹ-monophosphate, AX: reactance area, FEF_25__–__75%_: forced expiratory flow between 25% and 75% of FVC, FEV_1_: forced expiratory volume in 1 sec, FVC: forced vital capacity, ICS: inhaled corticosteroids, R5-R20: difference between the resistance of the respiratory system at 5 Hz and 20 Hz, R20: resistance of the respiratory system at 20 Hz, RV: residual volume, PC_20_: provocative concentration causing a 20% fall in FEV_1_, X5: reactance at 5 Hz.

### Adenosine dry powder tests

All four different adenosine dry powder tests were well tolerated by all subjects. A full dose release required a second inhalation for only 10% of the blisters. The technical test results with flow rate, inspiratory volume, and dose release are shown in Table 2.

**Table 2.** Technical results of adenosine dry powder test.

A. Peak and mean flow rates, and total volume attained per test							
Test specification		Peak inspiratory flow rate		Mean inspiratory flow rate		Total inspiratory volume	
Particle size (µm)	Inhalation flow (l min^–1^)	(l min^–1^)	(min to max)	(l min^–1^)	(min to max)	(l)	(min to max)
2.7	30–40	39.8	(35.1–46.4)	33.0	(28.9–40.4)	2.59	(0.70–4.95)
6.0	60–70	64.5	(60.3–72.1)	49.6	(41.3–53.3)	2.81	(0.57–5.03)
2.7	30–40	40.1	(35.1–46.1)	32.9	(29.1–38.6)	2.86	(0.60–5.52)
6.0	60–70	62.8	(57.0–70.9)	48.6	(44.2–52.3)	2.74	(0.72–3.95)

B. Number of incomplete adenosine dry powder releases after one inhalation per adenosine test				
Test specification				
Particle size (µm)	Inhalation flow (l min^–1^)	Number of provocation steps	2nd inhalation required	3rd inhalation required
2.7	30–40	82	9	0
6.0	60–70	73	6	0
2.7	30–40	94	17	5
6.0	60–70	90	1	0

*All patients were able to hold breath for 10 s*

### Differences in PD_20_ and PD_40_ threshold values between the four adenosine tests

**Figure 2.**
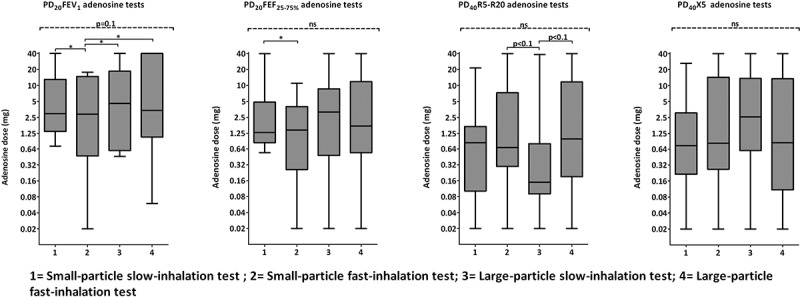
Box plots (25–75 percentile with range) of threshold values with differences between the four bronchoprovocation tests. PD_20_FEV_1_: adenosine dose causing a 20% fall in FEV_1_, PD_40_R5-R20: adenosine dose causing a 40% increase in R5-R20.

No significant differences were found between the four tests for the PD20FEV1 (p = 0.12), P20FEF25–75% (p = 0.37), PD40R5-R20 (p = 0.60) and PD40X5 (p = 0.46) (Table 3 Figure 2). Pairwise comparison showed a few differences between the tests, e.g. the small-particle slow-inhalation test induced a higher PD20FEV1 than the other three tests (p μ0.05).

We had hypothesized that small particles inhaled with a low flow rate would induce an increased response of the small airways. Analyses using a linear mixed effect model showed that only inspiratory flow rate had a significant effect on the response in R5-R20, i.e. a slow inhalation induced a lower PD40R5-R20 (Estimate -1.61, p=0.04). No other significant effect of particle size of inspiratory flow was found.

### Large and small airway response to adenosine tests

**Figure 3.**
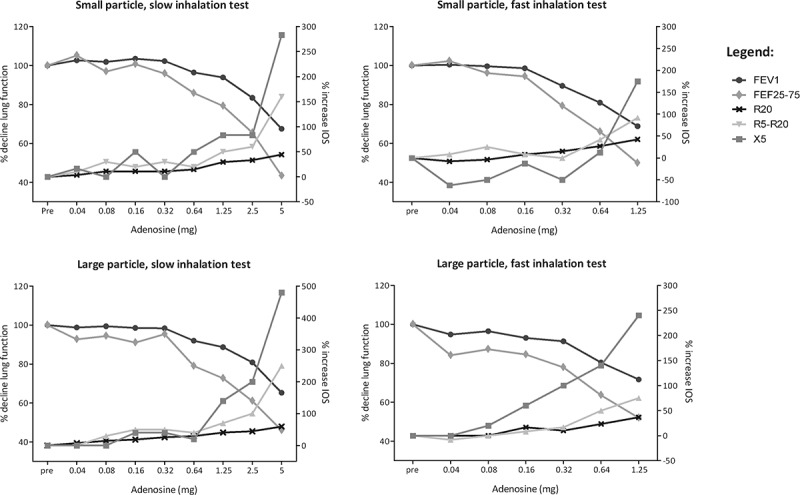
Typical example of large and small airway response per test.

The large and small airway response to the four adenosine tests are illustrated in Figure 3, showing one subject as typical example. The small-particle slow-inhalation test induced in the majority of the subjects responses in the parameters FEV_1_, FEF_25–75%_, R5-R20 and X5 (Table 4). A response in R20 was present in only 11 out of all 42 tests. As a result, PD_40_R20 was not included in further analyses. Noteworthy is the high response rate with small airway parameters induced by the large-particle tests, i.e. 8–10 out of 11 subjects.

**Table 3.** Threshold values of adenosine challenge with the four bronchoprovocation tests.

	**Small** particles, **slow** inhalation	**Small** particles, **fast** inhalation	**Large** particles, **slow** inhalation	**Large** particles, **fast** inhalation	*p*-value^#^
Large airways					
PD_20_FEV_1_ (mg)*	2.95^║^	2.49	4.62^║^	3.40^║^	0.12
Small airways					
PD_20_FEF_25–75%_ (mg)*	1.29	1.30	3.15	1.72	0.37
PD_40_R5-R20 (mg)*	0.83	0.68	0.15	0.98	0.60
PD_40_X5 (mg)*	0.74	0.82	2.59	0.84	0.46

*Values were log2 transformed. Median ^#^ *p*-value of Friedman test. Pairwise comparison of the four adenosine tests, results of linear mixed effect model. ^║^significant different from small-particle fast-inhalation test (*p *< 0.05).

PD_20_FEV_1_: adenosine dose causing a 20% fall in FEV_1_, PD_20_FEF_25–__75%_: adenosine dose causing a 20% fall in FEF_25–__75%_, PD_40_R5-R20: adenosine dose causing a 40% increase in R5-R20, PD_40_X5: adenosine dose causing a 40% increase in X5. Median of PD_40_R20 are not shown, due to the small sample size.

We had hypothesized that the four different adenosine tests would elicit the site of airway inflammation based on different response patterns, i.e. a response in the large airways only, in the small airways only, in the large and small airways, or no response in the large and small airways (Figure 1). However, none of the subjects showed a response exclusively of the large airways (Table 5).

**Table 4.** Responses per test.

*Percentage of subjects showing a response per test*		Small particles, slow inhalation (***n* = 10**)	Small particles, fast inhalation (***n* = 10**)	Large particles, slow inhalation (***n* = 11**)	Large particles, fast inhalation (***n* = 11**)
Large airways	20% decrease in FEV_1_	8	10	9	7
	40% increase in R20	2	2	1	3
Small airways	20% decrease in FEF_25–75%_	9	10	10	9
	40% increase in R5-R20	8	7	9	9
	40% increase in X5	8	7	9	8

**Table 5.** Response patterns.

*Subjects showing a response in the large and/or small airways*	Small particles, slow inhalation (***n* = 10**)	Small particles, fast inhalation (***n* = 10**)	Large particles, slow inhalation (***n* = 11**)	Large particles, fast inhalation (***n* = 11**)
Only a response in the large* airways	0	0	0	0
Only a response in the small** airways	1	0	2	2
Response in the large* and small** airways	9	10	9	8
No response in the large* or small** airways	0	0	0	1

*Large airway response based on ≥20% decrease in FEV_1_

**Small airway response based on ≥20% decrease in FEF_25–75%_, or ≥40% increase in R5-R20 or X5

## Discussion

This study shows that the adenosine dry powder challenge can provoke large and small airway constrictive responses in subjects with asthma. However, in contrast to our hypothesis we could not demonstrate that our small-particle slow-inhalation test provoked a more pronounced response in the small airways than the three other tests. Interestingly, all four dry powder adenosine tests, even the one with large particles at a fast inhalation, provoked a response in the small airways.

This is the first study showing that a bronchoprovocation test with a dry powder agent is able to induce a significant response in the large and small airways in subjects with asthma. With respect to the response rate this dry powder adenosine test is an improvement to the small- and large-particle nebulized AMP test used by Cohen and colleagues.[6] The use of an inhaler with an air classifier dispersion system is also totally new and enabled administration of a consistent aerosol in terms of particle size distribution over the total dose range.

We expected to observe a pronounced small and large airway response with the small-particle slow-inhalation test and a predominantly large airway response with the other combinations (Figure 1). However, almost every subject in our study demonstrated a response in both the large and small airways, independent of particle size and inhalation flow rate. How can we explain these unexpected results? First, it may be that we did not achieve the expected selective deposition of large particles in the large airways and small particles in the small airways. As we did not perform an imaging study with radio-labeled adenosine particles, we relied on the findings of Usmani and colleagues showing the differential deposition patterns of salbutamol.[4] We realize that the aerodynamic properties of adenosine may differ from salbutamol. Furthermore, the dry powder adenosine was not monodisperse even though the delivered adenosine aerosols had a relatively narrow size distribution. Possibly the use of monodisperse particles with a smaller diameter would have been more discriminating. A second explanation may be that the lung function tests used in this study have had limited specificity to discriminate between responses in the large and small airways. It has already been suggested that FEF_25–75%_ values not only reflect small airway dysfunction but also partly reflect large airway dysfunction.[19] With regard to IOS, we could not perform analyses with the R20, because this parameter demonstrated a response in only 26% of all tests, probably reflecting the poor ability of the cartilaginous central airways to narrow. Another explanation may be that deposition of adenosine in the large airways not only leads to obstruction in the large airways but also in the small airways. The small airways may also respond to inflammatory mediators transported distally via superficial capillary vessels, or to stimulation of sensory nerves with excitation of cholinergic reflex pathways. A neural mechanism has not extensively been investigated in human bronchial hyperresponsiveness yet, but this may be worthwhile in perspective of our findings.[20–22]

An important strength of this study is the use of the same inhaler in all four tests, in combination with a controlled inspiratory maneuver. By minimizing the factors that could affect the deposition of adenosine, we have been able to attribute our observations during the four tests to particle size and flow rate. A limitation of the study is that the bronchoprovocations were stopped based on the fall in FEV_1_, a large airway parameter. The increase in R5-R20, a small airway parameter, varied between 40 and 500% for the last given dose. We chose to stop the test at a 20% fall in FEV_1_ for safety reasons, as we had limited experience with dry powder adenosine, as well as with specifically provoking the small airways. We used threshold values of a 20% fall in FEF_25–75%_ and 40% increase in IOS parameters in our analyses. These are arbitrary cut-off values based on previous studies, using a threshold of 40% increase in resistance measured with the forced oscillation technique. We anticipate that a better insight in the small airway response can be obtained if bronchoprovocation continues to a predefined dose that is similar for all subjects. This enables a fair comparison of the tests and parameters between the subjects. Another limitation of this study is the small sample size and for this reason we cannot draw firm conclusions from our results.

To conclude, this study shows that a dry powder adenosine challenge is an appropriate test to induce bronchial hyperresponsiveness and can be readily used. All four dry powder adenosine tests, even the large-particle fast-inhalation test, provoked a response in the small airways. In our opinion, the next phase in the investigation of the small airways should be to elucidate the exact sites of adenosine deposition and bronchoconstrictor response, e.g. by using imaging techniques.

## Geolocation information

The research is performed in the University Medical Center Groningen, the Netherlands. N 53 13 15.4, E 6 34 37.7

## References

[1] Usmani OS, Biddiscombe MF, Barnes PJ. Regional lung deposition and bronchodilator response as a function of beta2-agonist particle size. Am J Respir Crit Care Med. 2005 12 15;172(12):1497–8. 16192448 10.1164/rccm.200410-1414OC

[2] Majoral C, Fleming J, Conway J, et al Controlled, parametric, individualized, 2D and 3D imaging measurements of aerosol deposition in the respiratory tract of healthy human volunteers: in vivo data analysis. J Aerosol Med Pulm Drug Deliv. 2014 1 8. 10.1089/jamp.2013.1065 24400875

[3] Price D, Martin RJ, Barnes N, et al Prescribing practices and asthma control with hydrofluoroalkane-beclomethasone and fluticasone: a real-world observational study. J Allergy Clin Immunol. 2010 9;126(3):511–518.e1–10. 10.1016/j.jaci.2010.06.040 20692026

[4] Cohen J, Douma WR, ten Hacken NH, et al Ciclesonide improves measures of small airway involvement in asthma. Eur Respir J. 2008 06;31(6):1213–1220. 18287130 10.1183/09031936.00082407

[5] van den Berge M, ten Hacken NH, van der Wiel E, et al Treatment of the bronchial tree from beginning to end: targeting small airway inflammation in asthma. Allergy. 2013 1;68(1):16–26. 23210509 10.1111/all.12062

[6] Cohen J, Postma DS, Douma WR, et al Particle size matters: diagnostics and treatment of small airways involvement in asthma. Eur Respir J. 2011 3;37(3):532–540. 20595155 10.1183/09031936.00204109

[7] Lexmond AJ, Hagedoorn P, Frijlink HW, et al Challenging the two-minute tidal breathing challenge test. J Aerosol Med Pulm Drug Deliv. 2013 3 19. 10.1089/jamp.2012.1021 23509936

[8] Lexmond AJ, Hagedoorn P, van der Wiel E, et al Adenosine dry powder inhalation for bronchial challenge testing, part 1: inhaler and formulation development and in vitro performance testing. Eur J Pharm Biopharm. 2014 1;86(1):105–114. 24140941 10.1016/j.ejpb.2013.06.027

[9] Oostveen E, MacLeod D, Lorino H, et al The forced oscillation technique in clinical practice: methodology, recommendations and future developments. Eur Respir J. 2003 12;22(6):1026–1041. 14680096 10.1183/09031936.03.00089403

[10] Miller MR, Hankinson J, Brusasco V, et al Standardisation of spirometry. Eur Respir J. 2005 08;26(2):319–338. 16055882 10.1183/09031936.05.00034805

[11] Wanger J, Clausen JL, Coates A, et al Standardisation of the measurement of lung volumes. Eur Respir J. 2005 09;26(3):511–522. 16135736 10.1183/09031936.05.00035005

[12] Sterk PJ, Fabbri LM, Quanjer PH, et al Airway responsiveness. Standardized challenge testing with pharmacological, physical and sensitizing stimuli in adults. Report Working Party Standardization of Lung Function Tests, European Community for Steel and Coal. Off Statement Eur Respir Soc. 1993 03;16:53–83. 8499055

[13] Crapo RO, Casaburi R, Coates AL, et al Guidelines for methacholine and exercise challenge testing-1999. This official statement of the American Thoracic Society was adopted by the ATS Board of Directors, July 1999. Am J Respir Crit Care Med. 2000 1;161(1):309–329. 10619836 10.1164/ajrccm.161.1.ats11-99

[14] de Boer AH, Hagedoorn P, Westerman EM, et al Design and in vitro performance testing of multiple air classifier technology in a new disposable inhaler concept (Twincer) for high powder doses. Eur J Pharm Sci. 2006 6;28(3):171–178. 16650739 10.1016/j.ejps.2005.11.013

[15] de Boer AH, Hagedoorn P, Gjaltema D, et al Air classifier technology (ACT) in dry powder inhalation. Part 1. Introduction of a novel force distribution concept (FDC) explaining the performance of a basic air classifier on adhesive mixtures. Int J Pharm. 2003 7 24;260(2):187–200. 12842339 10.1016/s0378-5173(03)00250-3

[16] Lexmond AJ, van der Wiel E, Hagedoorn P, et al Adenosine dry powder inhalation for bronchial challenge testing, part 2: proof of concept in asthmatic subjects. Eur J Pharm Biopharm. 2014; 88(1):148–152. 10.1016/j.ejpb.2014.04.008 24780441

[17] Broeders ME, Molema J, Hop WC, et al Bronchial challenge, assessed with forced expiratory manoeuvres and airway impedance. Respir Med. 2005 08;99(8):1046–1052. 15950147 10.1016/j.rmed.2005.01.006

[18] Weersink EJ, vd Elshout FJ, van Herwaarden CV, et al Bronchial responsiveness to histamine and methacholine measured with forced expirations and with the forced oscillation technique. Respir Med. 1995 05;89(5):351–356. 7638370 10.1016/0954-6111(95)90007-1

[19] Contoli M, Bousquet J, Fabbri LM, et al The small airways and distal lung compartment in asthma and COPD: a time for reappraisal. Allergy. 2010 2;65(2):141–151. 19909298 10.1111/j.1398-9995.2009.02242.x

[20] Pérez Fontán JJ, Kinloch LP, Donnelly DF Integration of bronchomotor and ventilatory responses to chemoreceptor stimulation in developing sheep. Respir Physiol. 1998 1;111(1):1–13. 9496467 10.1016/s0034-5687(97)00112-6

[21] Fontán JJ, Diec CT, Velloff CR Bilateral distribution of vagal motor and sensory nerve fibers in the rat’s lungs and airways. Am J Physiol Regul Integr Comp Physiol. 2000 8;279(2):R713–R728. 10938263 10.1152/ajpregu.2000.279.2.R713

[22] Lutz W, Sułkowski WJ Vagus nerve participates in regulation of the airways: inflammatory response and hyperreactivity induced by occupational asthmogens. Int J Occup Med Environ Health. 2004;17(4):417–431. 15852756

